# Isoniazid-Resistant Tuberculous Meningitis, United States, 1993–2005

**DOI:** 10.3201/eid1703.101715

**Published:** 2011-03

**Authors:** Christopher Vinnard, Carla A. Winston, E. Paul Wileyto, Rob Roy MacGregor, Gregory P. Bisson

**Affiliations:** Author affiliations: University of Pennsylvania School of Medicine, Philadelphia, Pennsylvania, USA (C. Vinnard, E.P. Wileyto, R.R. MacGregor, G.P. Bisson);; Centers for Disease Control and Prevention, Atlanta, Georgia, USA (C.A. Winston)

**Keywords:** Tuberculosis and other mycobacteria, bacteria, meningitis, epidemiology, drug resistance, isoniazid resistance, United States, dispatch

## Abstract

To determine patient characteristics associated with isoniazid resistance in cases of tuberculous meningitis, we conducted a cross-sectional study by using data from the US National Tuberculosis Surveillance System during 1993–2005. Foreign-born patients were more likely to be infected with an isoniazid-resistant strain.

The mortality rate for tuberculous meningitis (TBM) is higher than other forms of tuberculosis (TB), and survivors are often left with severe neurologic disability (*1*). We have recently shown that infection with isoniazid-resistant (rifampin-susceptible) *Mycobacterium tuberculosis* was associated with a 2-fold increase in the odds of death during therapy among patients with TBM who had positive cerebrospinal fluid (CSF) cultures, compared with patients with isoniazid-susceptible cases (*2*). When patients have a history of TB, clinicians may consider treatment history and drug susceptibilities in choosing empiric therapy (*3*). In contrast, little guidance is available to the clinician in the selection of an empiric regimen for patients without a history of treatment. Given that host and pathogen genotypes have been found to jointly influence the propensity of *M. tuberculosis* to cause meningeal infection, the epidemiology of isoniazid-resistant TBM may be different for meningeal and nonmeningeal forms of TB (*4*). We sought to determine the patient characteristics independently associated with isoniazid resistance on initial susceptibility testing among patients with TBM in the United States.

## The Study

We performed a cross-sectional study of clinical and demographic factors associated with isoniazid resistance on initial susceptibility testing in patients with TBM by using data from the United States National Tuberculosis Surveillance System. We examined data on all TB cases reported from January 1, 1993, through December 31, 2005. Patients were included if a clinical diagnosis of meningitis was made, positive cultures for *M. tuberculosis* were obtained from CSF, and results of any initial drug susceptibility testing were recorded. To study factors associated with isoniazid resistance in patients without a treatment history, we excluded patients with a previous diagnosis of TB. We also excluded patients with multidrug-resistant disease on the basis of evidence for differences in the epidemiology of isoniazid-resistant (rifampin-susceptible) and multidrug-resistant TB (*5*).

Differences in characteristics between the isoniazid-resistant and isoniazid-susceptible groups were assessed by using χ^2^ test and were selected for evaluation in a multivariable logistic regression model if unadjusted analysis demonstrated an association (p<0.25). An odds ratio (OR) for the association between a patient characteristic and initial isoniazid resistance was determined, along with its associated 95% confidence interval (CI). Multiple imputation was used to account for missing observations and permit complete data methods for analysis, under the assumption that missing data followed a missing-at-random pattern (*6*). Likelihood ratio testing was used to compare nested models, and the Akaike Information Criteria were used to compare non-nested models.

During 1993–2005, a total of 1,649 patients had a diagnosis of TBM, no previous history of TB, positive CSF cultures, and initial drug susceptibility testing. Of these 1,649 patients, 234 patients (14%) were infected with an isolate resistant to at least 1 first-line agent (isoniazid, rifampin, ethambutol, pyrazinamide, or streptomycin). Overall, 133 of 1,649 (8%) patients were infected with an isolate resistant to at least isoniazid.

After we excluded 11 patients without susceptibility testing results for isoniazid and 24 patients with multidrug-resistant disease, we compared 109 patients with at least isoniazid-resistant disease with 1,505 patients with isoniazid-susceptible disease. Unadjusted associations of clinical and demographic characteristics with initial isoniazid resistance are shown in Table 1. Foreign-born patients were more likely than US-born patients to have isoniazid-resistant disease, with an OR of 2.53 (95% CI 1.66–3.88). Overall, 849 of 1,614 (53%) patients in the primary analysis had a known HIV status, and 765 of 1,614 (47%) patients had unknown HIV status. Of the patients with known HIV status, 362 of 849 (43%) were HIV positive, and 487 of 849 were HIV negative (57%). HIV infection was not associated with initial isoniazid resistance (OR 1.10, 95% CI 0.62–1.95). Among HIV-positive patients, the association between foreign birth and initial isoniazid resistance was 3.05 (95% CI 1.54–6.06), and among HIV-negative patients it was 1.60 (95% CI 0.70–3.65).

**Table 1:** Unadjusted analysis of factors associated with initial isoniazid resistance in tuberculosis disease, United States, 1993–2005*

Patient characteristics	No. cases with isoniazid resistance/total no. cases (%)	OR (95% CI)	p value
Origin†			
US-born	40/926 (4)	Reference	
Foreign-born	69/674 (10)	2.53 (1.66–3.88)	<0.01
Age category, y			<0.01
<1	1/57 (2)	0.14 (0–0.86)	
1–<4	4/94 (4)	0.34 (0.08–1.00)	
4–<14	3/50 (6)	0.49 (0.09–1.67)	
14–<24	8/111 (7)	0.59 (0.23–1.38)	
24–<34	31/268 (12)	Reference	
34–<44	29/344 (8)	0.70 (0.40–1.24)	
44–<54	14/247 (6)	0.46 (0.22–0.92)	
55–<64	11/157 (7)	0.58 (0.25–1.22)	
64–74	5/159 (3)	0.25 (0.07–0.66)	
>74	3/127 (2)	0.18 (0.04–0.61)	
Race category			
White, non-Hispanic	14/240 (6)	Reference	0.03
Black, non-Hispanic	27/578 (5)	0.79 (0.39–1.66)	
Hispanic	38/489 (8)	1.36 (0.70–2.78)	
Asian/Native Hawaiian, non-Hispanic	28/276 (10)	1.82 (0.90–3.84)	
American Indian, non-Hispanic	0/16	0 (0–4.02)	
HIV status‡			
Negative	32/487 (7)	Reference	
Positive	26/362 (7)	1.10 (0.62–1.95)	0.73
Sex			
F	48/650 (7)	Reference	
M	61/964 (6)	0.85 (0.56–1.28)	0.41
Homeless within the previous year			
No	99/1,426 (7)	Reference	
Yes	3/74 (4)	0.57 (0.11–1.78)	0.48
Resident of a long-term care facility at diagnosis			
No	105/1,491 (7)	Reference	
Yes	2/67 (3)	0.41 (0.05–1.57)	0.20
Resident of a correctional facility at diagnosis			
No	106/1,561 (7)	Reference	
Yes	3/43 (7)	1.03 (0.20–3.32)	0.96
Pulmonary disease			
No	69/1,068 (6)	Reference	
Yes	40/546 (7)	1.14 (0.74–1.74)	0.51
Abnormal chest radiograph results			
No	48/690 (7)	Reference	
Yes	58/835 (7)	1.00 (0.66–1.52)	0.99
Positive smear (nonsputum site)			
No	71/985 (7)	Reference	
Yes	32/434 (7)	1.02 (0.64–1.61)	0.91
Positive tuberculin skin test result			
No	24/433 (5)	Reference	
Yes	36/520 (7)	1.33 (0.75–2.38)	0.30

*OR, odds ratio; CI, confidence interval. †US-born persons were defined as persons born in the United States, Puerto Rico, or US outlying area, or born abroad to American parents; all other persons were defined as foreign-born. ‡California reported only patients matched to the California AIDS registry from 1993–2004 as HIV positive; all other California patients are missing HIV status.

Based on unadjusted analyses, the multivariable model included age, race, residence in a long-term care facility, and foreign birth. Only foreign birth remained independently associated with initial isoniazid resistance (Table 2). Before age was adjusted for, the OR for foreign birth and initial isoniazid resistance was 2.53 (95% CI 1.66–3.88), and after adjusting for age, the OR was 2.25 (95% CI 1.47–3.43). Mexico was the most commonly reported country of origin for foreign-born patients, accounting for 20 of 69 foreign-born patients with isoniazid-resistant disease. Countries in Asia accounted for 7 of 9 countries with >2 cases of isoniazid-resistant TBM (Figure).

**Table 2:** Adjusted analysis of factors associated with initial isoniazid resistance in tuberculosis disease, United States, 1993–2005*

Characteristic	Adjusted OR (95% CI)	p value
Foreign-born	2.25 (1.47–3.43)	<0.01
Age category, y		0.10
<1	0.22 (0.03–1.70)	
1–<4	0.52 (0.17–1.57)	
4–<14	0.60 (0.17–2.06)	
14 –<24	0.53 (0.24–1.21)	
24–<34	Reference	
34– <44	0.80 (0.46–1.37)	
44–<54	0.54 (0.28–1.06)	
54 <64	0.60 (0.29–1.24)	
64–74	0.28 (0.11–0.74)	
>74	0.22 (0.07–0.74)	

*OR, odds ratio; CI, confidence interval.

**Figure:**
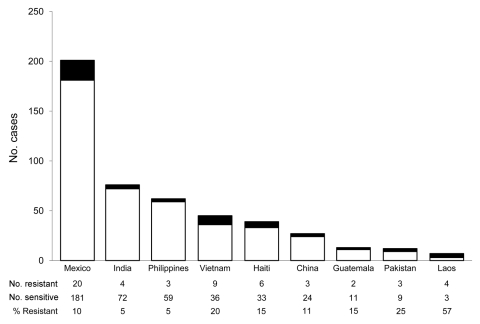
Countries of origin for foreign-born persons with tuberculous meningitis, United States, 1993–2005. Black bar sections indicate isoniazid-resistant and white bar sections isoniazid-sensitive tuberculosis.

## Conclusions

In this national cohort of patients with TBM, initial isoniazid resistance was more commonly seen in patients born outside the United States. In an earlier study of all forms of isoniazid monoresistant TB in the United States, foreign birth was also found to be a significant risk factor (*5*). Although we excluded patients with a diagnosis of TB, clinicians may have been unaware of a patient’s previous episode of TB that was treated before their arrival in the United States. Foreign-born patients may also have emigrated from countries with a higher prevalence of isoniazid resistance among newly diagnosed cases. Initial isoniazid resistance was also uncommon in persons >64 years of age. Older persons may have been exposed to TB antecedent to the use of isoniazid in treatment regimens, leading to reactivation with a drug-susceptible strain.

HIV infection is associated not only with increased risk for progression to active TB, but also an increased risk for extrapulmonary involvement among patients with active cases, including an increased risk for TBM (*7*). However, we did not see an association between HIV and initial isoniazid resistance among persons with known HIV status (p = 0.73), and the strength of the association between foreign birth and initial isoniazid resistance was not significantly modified by the presence of HIV infection. Similar to our findings, a lack of association between HIV and isoniazid resistance was seen in all cases of TB in the United States (with 13% known HIV positive) and the United Kingdom (with 5% known HIV positive) (*5**,**8*).

HIV status was missing for 47% of patients with TBM during the study period. For all states except California, reporting of HIV status to the National Tuberculosis Surveillance System increased from 36% in 1993 to 79% in 2008 (*9*). California reported only patients matched to the California AIDS registry during 1993–2004 as HIV positive. All other California patients are missing HIV status.

This study had several other limitations. Individual MIC levels for isoniazid were unavailable, and reporting does not distinguish between low-level and high-level resistance. Although we excluded patients with a history of TB, we were unable to identify patients previously treated for latent TB, which was shown to be associated with isoniazid monoresistance in patients for whom active TB subsequently developed (*10*). In conclusion, foreign-born persons with TBM who seek care in the United States were more likely to be infected with an isoniazid-resistant strain of *M. tuberculosis* compared with US-born persons, and persons >64 years of age were less likely to have an isoniazid-resistant infection than were persons 25–34 years of age. Prospective studies are needed to determine whether individual patient characteristics can guide the selection of TBM therapies and lead to an improvement in clinical outcomes.
